# Thousand-And-One Kinase 3 in Pancreatic Ductal Adenocarcinoma Stemness, Microenvironment and Therapy Resistance

**DOI:** 10.3390/ijms27146290

**Published:** 2026-07-15

**Authors:** James Tai, Hong He, Marcos V. Perini, Mehrdad Nikfarjam

**Affiliations:** 1 Faculty of Medicine, Dentistry and Health Sciences, The University of Melbourne, Parkville, VIC 3052, Australia; j.tai@student.unimelb.edu.au (J.T.); marcos.perini@unimelb.edu.au (M.V.P.); m.nikfarjam@unimelb.edu.au (M.N.); 2 Department of Surgery, Austin Health, Heidelberg, VIC 3084, Australia

**Keywords:** thousand-and-one kinase, pancreatic ductal adenocarcinoma, targeted therapy, tumor, cancer stem cells, cachexia, tumor microenvironment

## Abstract

Pancreatic ductal adenocarcinoma (PDA) remains one of the most recalcitrant solid tumors, characterized by a fibrotic and immunosuppressive tumor microenvironment (TME), inherent chemoresistance and systemic cachexia. Recent pan-cancer proteomic and transcriptome analyses have identified Thousand-and-one amino acid kinase 3 (TAOK3) as a central signaling node governing these lethal hallmarks. Operating as a MAP kinase kinase kinase (MAP3K) within the STE20 family, TAOK3 coordinates diverse cellular processes. In the PDA parenchyma, TAOK3 maintains the cancer stem cell (CSC) phenotype by enforcing a strict DNA damage response checkpoint. Within the TME, TAOK3 facilitates local invasion by regulating endosomal trafficking for invadopodia assembly. Furthermore, TAOK3 exerts immunomodulatory effects, dictating macrophage polarization and preserving canonical T-cell receptor (TCR) signaling by inducing the degradation of the SHP-1 phosphatase. Systemically, tumor-derived signals establish a pathogenic feedforward loop with skeletal muscle whereby TAOK1 activity is upregulated in peripheral skeletal muscle to drive muscle atrophy secondary to TAOK3 expression in the primary tumor. The recent development of specific structural inhibitors with dual-acting natural bioactivities, provides a compelling preclinical foundation to transition TAOK3 from prognostic biomarker to premier therapeutic target in pancreatic oncology.

## 1. Introduction

Pancreatic ductal adenocarcinoma (PDA) represents one of the most formidable malignancies in contemporary oncology, characterized by a near-uniform mortality rate and an exceptionally poor prognosis [1,2,3]. Epidemiological data indicate that pancreatic cancer causes almost as many deaths annually as there are incident cases in developed countries [1,4]. In the GLOBOCAN statistics from 2022, pancreatic cancer was ranked twelfth for incidence but sixth for cancer-related mortalities worldwide [5]. A complex interaction of modifiable risk factors including obesity, diabetes, alcohol consumption, smoking, and chronic pancreatitis with non-modifiable genetic predispositions governs the pathophysiology of PDA [1,4,6].

At the molecular level, the pathogenesis of PDA is fundamentally driven by activating point mutations in the *KRAS* oncogene, present in over 90% of early precursor lesions, including pancreatic intraepithelial neoplasia (PanIN) [1,7]. This oncogenic event is followed by the catastrophic loss or mutational inactivation of critical tumor suppressor genes, including *TP53*, *CDKN2A*, and *SMAD4* [8,9]. The disease typically exhibits a progressive developmental pathway from normal ductal epithelium through successive grades of PanIN (1A, 1B, 2, and 3) to fully invasive ductal carcinoma, though it may also originate from the malignant transformation of an intraductal papillary mucinous neoplasm (IPMN) or mucinous cystic neoplasm [10].

The clinical management of PDA is encumbered by its insidious onset and often late clinical presentation. The disease is fundamentally understood to be a systemic, micrometastatic disease from early inception [11]. As such, patients frequently present with advanced, locally invasive, or metastatic disease rendering them non-operative candidates in 80–90% of cases [4,12,13]. For the highly selected subset of patients who are candidates for upfront surgical resection, long-term outcomes remain disheartening [14]. It is increasingly recognized that long-term survival depends strictly on favorable tumor biology and achieving a true R0 resection [13,15].

Standard-of-care pharmacotherapy for PDA has evolved slowly. Gemcitabine, established as the reference first-line therapy in 1997, provides negligible long-term survival benefits over 5-fluorouracil (5-FU) [11]. The introduction of multi-agent regimens, specifically FOLFIRINOX (5-FU, leucovorin, irinotecan, and oxaliplatin) and the combination of nab-paclitaxel (Abraxane) with gemcitabine, has demonstrated superiority in improving median survival to 11.1 months and 8.5 months, respectively [11,16]. However, these aggressive regimens are accompanied by substantial systemic toxicity, restricting their use to patients with excellent performance status [7,11]. Precision medicine and targeted therapies have also yielded limited success in PDA. To date, the EGFR inhibitor erlotinib remains the only targeted agent FDA-approved in combination with gemcitabine, though it provides only a fractional survival benefit [11]. Furthermore, contemporary clinical trials investigating immunotherapies including anti-PD-1/PD-L1 and anti-CTLA-4 checkpoint inhibitors, GVAX vaccines, adoptive T-cell therapies, and molecular inhibitors of the MAPK/PI3K, JAK-STAT, Hedgehog, and Notch pathways have largely failed to overcome the profound therapeutic resistance inherent to PDA [7,11]. More recently, direct pharmacological targeting of the KRAS oncoprotein has emerged as parallel strategy in PDA [17]. Mutant-selective inhibitors of KRAS G12C (sotorasib, adagrasib) and the KRAS G12D-selective agent MRTX1133 have entered clinical and late preclinical evaluation, while the multiselective RAS(ON) inhibitor daraxonrasib (RMC-6236) has produced objective responses in a subset of patients with previously treated RAS-mutated PDA in a recent phase 1–2 trial [17,18]. However, clinical responses to KRAS G12C inhibitors in PDA remain modest, and resistance to RAS-directed agents frequently emerges through feedback reactivation of parallel signaling nodes [17,18]. These findings indicate that RAS-directed monotherapy is unlikely to be curative in PDA, reinforcing the rationale for identifying complementary, non-RAS actionable nodes, such as TAOK3, that operate downstream to or in parallel with oncogenic KRAS signaling.

This therapeutic resistance of PDA is heavily predicated on the unique tumor microenvironment (TME) [9,19]. PDA is characterized by a dense, fibrotic stromal reaction comprising up to 90% of tumor volume [9]. High concentrations of glycosaminoglycans within this stroma generate extreme interstitial pressures that compress the local vasculature [9]. This vascular collapse not only impedes the delivery of cytotoxic drugs but establishes a hypoxic, nutrient-deprived immunosuppressive niche that restricts the infiltration and activation of anti-tumor immune cells [20,21]. Experimental strategies attempting to dismantle this barrier, such as the use of PEGPH20 to deplete stromal hyaluronic acid, underscore the necessity of dual-targeting the tumor parenchyma and its surrounding microenvironment [11,19].

Compounding this therapeutic recalcitrance is the profound molecular heterogeneity intrinsic to PDA [22]. Transcriptomic profiling of primary tumors has resolved PDA into molecular subtypes; most consistently a ‘classical’ and a ‘basal-like’/’squamous’ phenotype, with additional ‘quasimesenchymal’, ‘exocrine-like’, ‘progenitor’, ‘immunogenic’, and ‘aberrantly differentiated endocrine exocrine’ (ADEX) [22,23,24]. These subtypes differ in stromal composition, chemosensitivity, and prognosis [23]. Single-cell and genomic evolution studies indicate that individual tumors frequently harbor a continuum of these states rather than a single fixed identity, with ongoing clonal evolution reshaping this phenotypic landscape during disease progression and metastatic spread [25]. Any molecular node proposed as a driver of PDA lethality, including TAOK3, must, therefore, be interpreted against this backdrop of subtype- and lineage-specific heterogeneity. The pan-cancer and pan-PDA correlative analyses summarized below do not resolve whether TAOK3 dependency is uniform across the classical/basal-like-spectrum or whether TAOK3 itself contributes to determining subtype identity, and this remains an important open question for future subtype-stratified functional studies.

To breach the current therapeutic plateau, contemporary oncology must identify novel molecular nodes that govern tumor plasticity, therapy resistance, and the reciprocal signaling between the tumor and host. Comprehensive pan-cancer analyses have highlighted the Thousand-and-one amino acid (TAO) kinases, specifically TAOK3, as prognostic biomarkers and multifaceted regulators of cancer stem cell (CSC) maintenance, DNA damage repair, immune signaling, and systemic cachexia [26,27,28,29]. This review synthesizes the structural biology, oncogenic signaling paradigms and pathophysiological roles of TAOK3, establishing the fundamental biological rationale for its therapeutic targeting in pancreatic malignancies.

## 2. Structural Biology of TAOK3

The mammalian kinome encompasses an array of regulatory enzymes responsible for transducing extracellular stimuli into precise intracellular biological responses [30]. Pan-cancer proteomic and transcriptomic analyses, including The Cancer Genome Atlas, reveal that the kinome landscape is profoundly altered in malignancy [31,32]. The sterile (STE) group of kinases, fundamentally involved in mitogen-activated protein kinase (MAPK) cascades includes the highly specialized TAO kinase subfamily; TAOK1, TAOK2, and TAOK3 [30,33]. These kinases are encoded by distinct genetic loci across the human genome (Table 1) [34].

**Figure 1:**
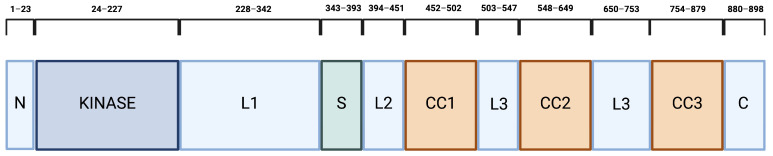
Diagrammatic representation of molecular structure of TAOK3 highlighting key functional domains and linking regions. N: N-terminal; KINASE: kinase domain; S: serine domain; L: linked region; CC: coiled-coil domain; C: C-terminal tail.

Each TAOK member features an N-terminal kinase domain that dictates their catalytic activity (Figure 1) [28]. This N-terminal catalytic region encompasses eleven canonical subdomains, featuring the STE20 signature sequence (GTPY/FWMAPE) located in subdomain VIII [35]. The kinase domains display extraordinary sequence homology; human TAOK3 shares an 88.6% and 82.7% structural identity with the kinase domains of TAOK1 and TAOK2, respectively [36].

In TAOK3, this N-terminal catalytic region encompasses all eleven canonical subdomains characteristic of classical serine/threonine kinases [35]. Extensive mutational profiling has revealed that the residues Threonine-181 and Tyrosine-183 within this specific signature sequence are absolute requirements for the catalytic functionality of TAOK3 [35,36].

Transitioning away from the N-terminus, the structural organization of the TAO kinases diverges to accommodate specific regulatory functions. Following the kinase domain, the proteins feature a highly conserved serine-rich domain situated approximately between amino acids 344 and 393 [28]. This is succeeded by a vastly different C-terminal non-catalytic regulatory region [37]. In TAOK3, this extensive domain encompasses amino acids 285 through 898 and contains three prominent coiled-coil regions [28]. This C-terminal region of TAOK3 is hypothesized to function as an molecular interaction hub, recruiting specific protein phosphatases and diverse interacting molecules necessary to modulate downstream substrate targeting and govern precise subcellular localization [35].

The biological regulation of TAOK3’s catalytic activity presents a unique paradigm within the STE20 family. While the majority of STE20 kinases activate stress-associated pathways such as the JNK/SAPK cascade, TAOK3 functions to inhibit the basal activity of JNK/SAPK [35,38]. Additionally, TAOK3 displays a highly distinctive and critical response to upstream growth factor signaling. It is uniquely regulated by the epidermal growth factor (EGF) receptor; following EGF stimulation, the intrinsic kinase activity and autophosphorylation levels of TAOK3 experience a dramatic and rapid reduction [30,39]. This specialized regulatory behavior suggests that TAOK3 operates strategically downstream of the EGF receptor but upstream of critical small GTPases such as Rac1 and Cdc42, positioning it as a fundamental control for cellular stress responses [35].

A high-resolution (1.50 Å) X-ray crystal structure of the human TAOK3 kinase domain in complex with its physiological ligand ADP has been deposited in the Protein Data Bank (PDB: 6BDN) and was utilized as the docking template for structure-based virtual screening that identified the TAOK3-directed lead Compound Z1 [40]. The TAOK3 structure confirms that TAO-family kinases adopt the canonical bilobal fold shared with other STE20-group kinases, in which activation-loop phosphorylation is expected to stabilize a conformation able to engage MEK3/MEK6 [33,40]. The TAOK3 structure defines an ATP-binding pocket bounded by the hinge region (including Met101 and Cyst104) and a hydrophobic sub pocket (including Val38 and Lys53) that has been directly exploited for inhibitor design [40]. The deposited TAOK3 structure captures a nucleotide (ADP)-bound state, and a structure resolving the unphosphorylated conformation analogous to that described for other STE20-group members is yet to be reported and remains an important target for future structural work [33].

## 3. TAOK3 Signaling Pathways

The physiological functions of the TAOK family span an array of fundamental cellular processes [28]. When dysregulated, these kinases heavily influence the trajectory of malignant transformation, operating as complex, context-dependent modulators of tumorigenesis across a spectrum of human cancers [26]. Comprehensive pan-cancer transcriptomic and genomic analyses reveal that TAOK3 does not adhere to a limited functional classification but rather assuming a dual role, acting as either a potent oncogene or a tumor suppressor depending on the specific tissue lineage and the established tumor microenvironment [26].

TAOK3 is established as an oncogenic driver in PDA as well as cholangiocarcinoma, hepatocellular carcinoma, gastric adenocarcinoma and esophageal squamous cell carcinoma [26,28,41]. Conversely, profiling of other malignancies indicates a potential tumor-suppressive role; TAOK3 expression is reduced in breast adenocarcinoma, colon adenocarcinoma, and lung adenocarcinoma [27,42]. The prognostic significance of TAOK3 is equally inconsistent. While elevated expression of TAOK3 correlates with favorable overall survival in renal clear cell carcinoma, it predicts substantially lower survival rates and aggressive disease progression in mesothelioma and esophageal squamous cell carcinoma [27].

**Figure 2:**
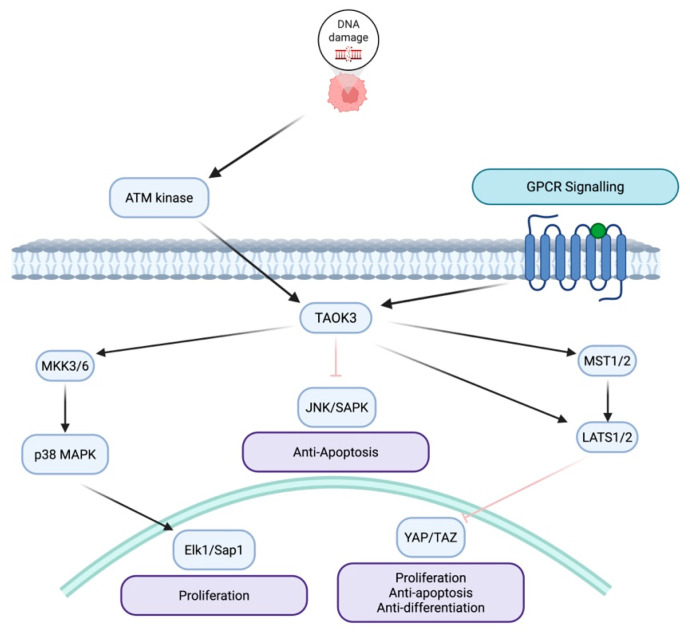
Schematic representation of the key intracellular signaling pathways of TAO kinases and respective cellular consequences. ATM; Ataxia-Telangiectasia Mutated, MKK; Mitogen-activated protein kinase, TAOK3; Thousand-and-one kinase 3, MAPK; Mitogen-activated protein kinase, JNK; c-Jun N-terminal kinase, SAPK; Stress-activated protein kinase, MST; Mammalian Ste20-like kinase, LATS; Large tumor suppressor kinase, YAP; Yes-activated protein, TAZ; Transcriptional co-activator with PDZ-binding motif, GPCR; G-protein-coupled receptor.

The molecular mechanisms underwriting TAOK3-driven tumorigenesis are linked to its capacity to operate as a MAP3K and integrate diverse signal transduction networks [26]. The primary signaling cascades governed by TAOK3 include the p38 MAPK pathway, SAPK/JNK pathway, and Hippo Signaling pathways (Figure 2).

### 3.1. p38 MAPK Pathway

TAO kinases serve as intermediates connecting upstream signals, such as genotoxic DNA damage or the activation of G-protein-coupled receptors (GPCRs), to the execution of the p38 MAPK cascade [28]. Signaling initiated by muscarinic agonists stimulate the G-alpha heterotrimeric G-protein consequently activating TAO kinases [43]. The active TAOK3 phosphorylates the MAP/ERK kinases MEK3 and MEK6, which then directly phosphorylate and activate p38 MAPK [28]. Activated p38 translocates to the nucleus to phosphorylate ternary complex factors like Sap 1 and Elk1, thereby driving the transcription of genes requisite for survival and proliferation [43].

### 3.2. SAPK/JNK Pathway

While the majority of STE20 kinases activate the stress-associated pathways, TAOK3 uniquely functions to inhibit the basal activity of the JNK/SAPK cascade [38]. This negative regulatory pathway is itself negatively regulated by EGF stimulation [39]. Knockdown modelling of TAOK3 has been shown to lead to JNK1/2 phosphorylation, activating Caspase-9 and triggering poly(ADP-ribose) polymerase cleavage, culminating in cellular apoptosis [27]. This would indicate that high baseline levels of TAOK3 protect neoplastic cells from spontaneous apoptotic death [44,45].

### 3.3. Hippo Signaling Pathway/YAP/TAZ Pathway

The Hippo pathway is a master regulator of organ size, cellular proliferation, stem cell function, and tissue regeneration [46,47,48]. Under normal physiological conditions, TAOK3 exerts upstream regulatory control by phosphorylating the activation loop of the MST1/2 kinases, or by directly acting on the downstream LATS1/2 kinases [44,47]. This coordinated signaling cascade ultimately leads to the phosphorylation, cytoplasmic sequestration, and subsequent proteasomal degradation of the potent oncogenic transcriptional co-activators YAP and TAZ [44,49]. In the context of PDA, the dysregulation of this TAOK3-driven Hippo axis leads to the aberrant nuclear accumulation of YAP/TAZ, driving rampant cellular proliferation, anti-apoptotic survival, and de-differentiation of epithelial cells [28,44].

### 3.4. Mitotic DNA Integrity Checkpoints

TAOK3 is a central component of a highly conserved network of 16 mitotic DNA integrity checkpoint kinases, functioning alongside well-characterized guardians of the genome such as ATM, CHEK1, CHEK2, CDK1, and PLK1 [34]. In malignant contexts, these kinases are indispensable for maintaining genomic stability in the face of rapid, error-prone proliferation and external genotoxic insults [34].

Genetic alterations within the *TAOK3* gene itself heavily influence tumor biology. Copy number variations are the most prevalent form of genetic alteration affecting *TAOK3* across the malignancy spectrum [26]. *TAOK3* mutations also demonstrate a profound positive correlation with the upregulation of canonical hallmark cancer genes, notably *SMAD2*, *SMAD4*, and *RNF168*, linking TAOK3 dysfunction directly to aberrant TGF-beta signaling, deregulated cell cycle progression, and defective DNA repair [26].

## 4. TAOK3 in Pancreatic Cancer Biology

TAOK3 is an oncogenic driver for PDA based on associative pan-cancer expression, mutational and phosphorylation-correlation analyses, although there is a paucity of direct interventional evidence [26]. Detailed comparative transcriptomic analyses of human cohorts consistently demonstrate a significant upregulation of TAOK3 mRNA transcripts and functional protein levels in pancreatic tumor specimens when compared with matched adjacent normal pancreatic tissues [26,50]. In more advanced stages of pancreatic cancer, the S331 locus of the TAOK3 protein is also subject to significantly higher levels of phosphorylation [26]. The loss- and gain-of-function data supporting a more direct, functionally necessary role for TAOK3 in PDA stemness and chemoresistance are presented below.

TAOK3 expression levels in PDA demonstrate a profound positive correlation with tumor mutational burden and are intimately linked with the immunological landscape of the tumor [26]. High TAOK3 expression correlates with elevated immune cell infiltrates, specifically mapping to the presence of activated CD4 T-cells, CD8 T-cells, type 2 T helper (Th2) cells, and neutrophils [26]. This correlation suggests that TAOK3 signaling is tied to the broader inflammatory and immune-evasive characteristics defining the PDA microenvironment.

### TAOK3 and Cancer Stem Cell Phenotype

The notorious intractability, metastatic dissemination and high recurrence rates defining PDA are fundamentally driven by a highly resilient subpopulation of cells existing within the tumor bulk [27,48]. These specialized cells are classified as cancer stem cells (CSCs) or tumor-initiating cells (TICs) [51]. Possessing augmented self-renewal capabilities, CSCs are the primary biological engines of tumor initiation and demonstrate intrinsic resistance to standard cytotoxic regimens, such as gemcitabine [9,52].

The precise identification and isolation of pancreatic CSCs rely on tracking distinct molecular surface markers and functional cellular phenotypes [53]. While substantial heterogeneity exists across different PDA cell lines, key established markers include c-Met, CD44, CD24, CD133, and CD326 (EpCam) [7,54]. The critical nature of these cells has driven the development of advanced qualitative high-throughput screening of extensive oncology libraries to identify CSC-specific inhibitors [55].

Emerging evidence positions TAOK3 as a central signaling pillar responsible for the maintenance and survival of the pancreatic CSC phenotype [56]. This role is supported by convergent loss-of-function and gain-of-function evidences. Genetic knockdown or pharmacological inhibition of TAOK3 selectively induces apoptosis in CSC-enriched spheroids without comparably affecting bulk monolayer cultures, while enforced TAOK3 overexpression is sufficient to induce a stem-like transcriptional and phenotypic shift [53,57]. This is evidenced by the pronounced transcriptional upregulation of core pluripotency mediators and established stemness transcription factors, including SOX2 and NANOG, as well as the surface expression of the canonical CSC markers CD44, CD24, and c-Met [56,57]. Consequently, cells harboring high TAOK3 levels exhibit aggressively augmented anchorage-independent growth, superior colony-forming capacities, and an enhanced ability to initiate primary tumor and establish liver metastases in vivo [50]. Together, these data indicate that TAOK3 is both necessary and, at least in overexpression contexts, sufficient to promote the CSC phenotype in the model systems examined [50,53,56,57].

The molecular mechanism by which TAOK3 preserves the CSC state is established in the regulation of the DNA Damage Response (DDR) and the maintenance of genomic integrity under severe stress [53]. Highly invasive pancreatic CSCs exhibit a significantly amplified baseline expression of DNA repair genes [51]. TAOK3 mediates the ATM-induced activation of the p38 signaling pathway [54]. This robust, pre-activated DDR machinery enables pancreatic CSCs to detect and resolve DNA double-strand breaks at an accelerated rate when challenged with potent genotoxic agents like gemcitabine [51]. In response to severe genotoxic stress, TAOK3 mediates the ATM-induced activation of the p38 signaling pathway through direct phosphorylation of the intermediate kinases MKK3 and MKK6 [53]. The targeted activation of the TAOK3-MKK3/6-p38 axis enforces a strict, impenetrable G2-M cell-cycle checkpoint [56]. By completely halting the cell cycle at the G2/M transition, TAOK3 ensures that the heavily damaged CSCs possess adequate time to resolve lesions before progressing into mitosis, thereby averting catastrophic chromosomal fragmentation and subsequent apoptosis [56].

**Figure 3:**
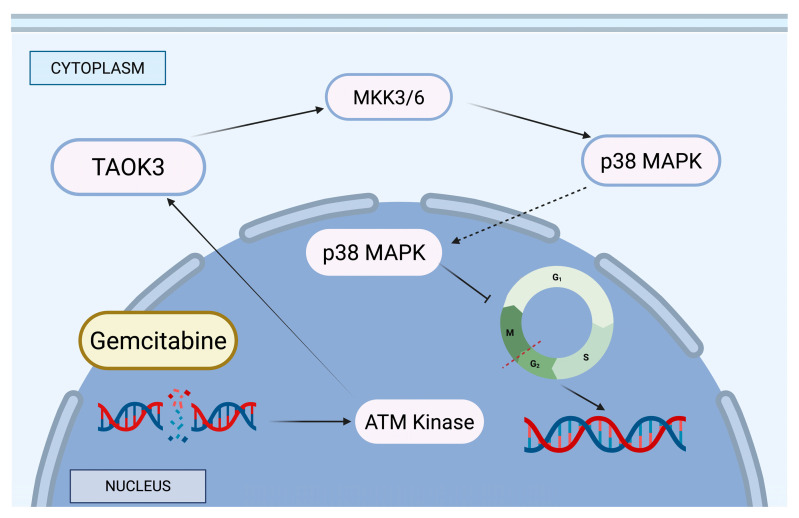
TAOK3-driven chemoresistance to gemcitabine via control of G2/M DNA repair checkpoint. Gemcitabine-induced DNA damage in tumor cells stimulates ATM kinase leading to TAOK3 activation and subsequent cascade leading to activation of the p38 MAPK pathway and arrest of the cell cycle at G2-M to facilitate DNA damage repair of for the tumor cell and persistent survival. Black arrows reflect pathway activations. Dashed black arrow reflects the cytoplasmic and nuclear elements of the p38 MAPK pathway. Dashed red line reflects the point of arrest of the cell cycle. ATM; Ataxia-Telangiectasia Mutated, TAOK3; Thousand-and-one kinase 3, MKK; Mitogen-activated protein kinase, MAPK; Mitogen-activated protein kinase.

The critical therapeutic vulnerability of this system lies in the concept of synthetic dependency. Pancreatic CSC-enriched spheroids exhibit a profound, selective reliance on TAOK3 signaling that is absent in the bulk tumor cell population [53]. It is hypothesized that while highly differentiated, non-stem monolayer cells often possess parallel compensatory signaling pathways capable of bypassing TAOK3 to manage genomic lesions, the stressed physiological state of the CSC population leaves them operating with a critically limited reserve to cope with interferences in their DDR network [53]. Consequently, the targeted genetic depletion or specific pharmacological inhibition of TAOK3 selectively and completely disrupts this protective mechanism in CSCs. Stripped of the TAOK3-mediated G2-M checkpoint, the CSCs accumulate unresolvable DNA double-strand breaks that overwhelm their limited reserves, forcing the entire stem cell population into apoptosis [53]. Through this mechanism, the blockade of TAOK3 effectively dismantles the primary engine of chemoresistance, re-sensitizing the recalcitrant PDA spheroids to standard therapies like gemcitabine and drastically reducing the long-term potential for metastatic recurrence (Figure 3) [56].

## 5. TAOK3 in the Tumor Microenvironment and Immune Response

The immunological and stromal compartments of PDA dictate disease progression in conjunction with the genetic mutations within the epithelial parenchyma [21]. A high CD4+/CD8+ T-cell ratio, and the presence of highly functional effector T-cells, have significant prognostic implications for patient survival [58].

### 5.1. Modulation of Canonical TCR Signaling and SHP-1 Degradation

Recent mechanistic studies have unveiled that TAOK3 is critical for canonical T-cell receptor (TCR) signaling and maintenance of T-cell responsiveness within the hostile TME [59]. Upon initial TCR engagement by an antigen-MHC complex, the rapid activation of the Src-family kinase LCK is required to initiate all downstream T-cell responses [60]. However, this LCK activation simultaneously triggers a potent negative feedback loop mediated by the tyrosine phosphatase SHP-1, which normally functions to dephosphorylate and terminate the TCR signal, preventing overactivation [60].

TAOK3 acts as a positive regulator of this process to ensure sustained T-cell activation [60]. Specifically, TAOK3 directly phosphorylates Threonine-394 located within the phosphatase domain of SHP-1 [60]. This promotes the ubiquitination and proteasomal degradation of SHP-1 thereby relieving this negative regulatory brake on LCK and sustaining TCR signal transmission; conversely, genetic loss of TAOK3 increases SHP-1 abundance and produces the opposite phenotype, desensitizing the TCR and impairing T-cell proliferation, IL-2 secretion and naïve T-cell survival [59,60]. As this mechanism sustains T-cell responsiveness, its net contribution to anti-tumor immunity in PDA likely depends on whether TAOK3 activity in tumor-infiltrating T-cells is preserved, reduced, or otherwise altered by the immunosuppressive TME, which is yet to be directly examined. We, therefore, do not classify this T-cell-intrinsic axis as an immune-evasion mechanism but rather predict that loss or reduction of TAOK3 activity within tumor-infiltrating T-cells, rather than its presence would favor immune evasion by desensitizing the TCR of tumor-reactive lymphocytes. With the expression of TAOK3 in T-cells within the PDA TME an open question, this has direct therapeutic implications, given TAOK3 inhibition could plausibly impair rather than restore protective T-cell responsiveness.

### 5.2. Regulation of Macrophage Polarisztion

TAOK3 orchestrates macrophage polarization, a key driver of both the fibrotic TME and chronic pancreatitis, a well-established inflammatory precursor to PDA [56,61]. During tissue damage, the release of damage-associated molecular patterns, including the calcium binding-protein S100A9, exploits the TAOK3-JNK signaling pathway to manipulate local inflammatory cells, which is a recognized mechanism of immune evasion in PDA [56]. Specifically, this signaling axis orchestrates the migration, recruitment and adhesion of macrophages into the damaged tissue, forcing their polarization into the highly immunosuppressive and pro-fibrotic M2-subtype [56]. Once polarized, these M2 macrophages establish pathogenic paracrine signaling with resident pancreatic stellate cells, coercing them into a state of hyperactivity resulting in severe fibrosis and the dense structural desmoplasia hallmark of PDA [56]. In the context of chronic pancreatitis, the targeted inhibition of S100A9 has been shown to significantly improve the fibrotic deposition by disrupting this exact TAOK3-mediated M2 polarization network [56].

### 5.3. Desmoplasia and Local Invasion

Within the tumor stroma, navigating the dense extracellular matrix requires specialized cellular machinery [62]. Invasive PDA cells develop actin-rich membrane protrusions, known as invadopodia, which orchestrate the localized proteolytic degradation of the surrounding matrix [37,63]. TAOK3 is a recognized regulator of the endosomal trafficking required to build the invadopodia [37]. TAOK3 localizes to RAB11-positive recycling endosomes and directly phosphorylates the dynein subunit LIC2 at the Threonine-202 residue, which drives the transport of these endosomes to deliver the scaffold protein TKS5a directly to the plasma membrane [37]. This is an absolute requirement for the physical assembly of invadopodia and subsequent vascular invasion.

## 6. TAO Kinases in Pancreatic Cancer Cachexia

Cancer cachexia is a devastating, multifactorial paraneoplastic syndrome that fundamentally results from an irreversible negative energy balance driven by profound systemic inflammation and aberrant host–tumor crosstalk [64,65]. The molecular basis of cachexia is driven by metabolic adaptations that prioritize tumor homeostasis and growth at the expense of host tissue preservation [65]. It is characterized by the rapid, involuntary loss of skeletal muscle mass and adipose tissue, impacting functional status, tolerance of systemic therapies and directly accounts for up to 20% of all cancer-related mortalities [66].

### The CCL2-TWEAK-TAOK1 Feedforward Muscle Atrophy Loop

In PDA, primary tumor cells and infiltrating tumor-associated macrophages (TAMs) engage in a directly demonstrated paracrine feedforward loop that drives systemic muscle wasting [67]. Tumor-derived CCL2 recruits CCR2-positive monocytes and polarizes them into TAMs; these TAMs secrete CCL5, which acts on tumor cell CCR5 to activate TRAF6-dependent canonical NF-kB (p65) signaling and to induce transcription of the cytokine TWEAK [67]. Genetic CCR2 deletion and CCL5/CCR5 blockade each attenuate circulating TWEAK, non-canonical NF-kB activation in skeletal muscle, induction of the muscle-specific E3 ubiquitin ligases MuRF1 and Atrogin-1, and muscle wasting in orthotopic murine PDA models in vivo, directly establishing this tumor-TAM-muscle relay as a genuine driver of PDA-associated cachexia [67].

A parallel, muscle-intrinsic axis converges on the paralogous kinase TAOK1 [68]. Exposing myotubes to tumor-conditioned medium of the MIA PaCa-2 cell line, or inflammatory mediators TNF- α and IL-6, activates TAOK1, which signals through p38-MAPK to drive nuclear translocation of FoxO3 and consequent induction of both the ubiquitin–proteasome system and the autophagy–lysosome system, the two principal effector pathways of cancer-associated muscle proteolysis [64]. Genetic knockdown or pharmacological inhibition of TAOK1 confers resistance to this atrophy in myotubes [68,69]. Yu et al. [50] showed that Corylifol A, a dual TAOK3/TAOK1 inhibitor, reduces both tumor burden and circulating TWEAK in MIA PaCa-2 xenograft-bearing mice while simultaneously suppressing TAOK1 activation and muscle atrophy in the same animals, providing the strongest PDA-specific evidence that tumor-derived and TAOK3-dependent signals converge functionally on TAOK1 in muscle. A direct mechanistic link from TWEAK/Fn14 engagement to TAOK1 activation has not been demonstrated; hence the TWEAK/Fn14 and TAOK1 arms should be regarded as convergent but mechanistically distinct contributors to cachexia.

**Figure 4:**
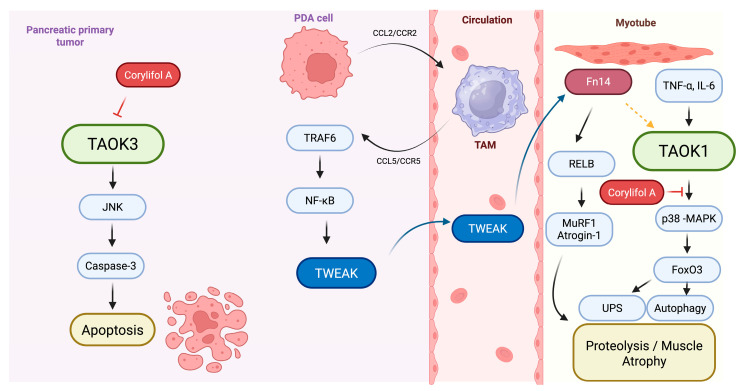
Proposed model of PDA-associated cachexia integrating TAOK3 prevention of PDA cell apoptosis, tumor–macrophage crosstalk with TAOK1-dependent muscle proteolysis. Solid arrows denote relationships directly demonstrated in PDA-relevant models, dashed arrows denote a proposed correlative link. Red bars denote pharmacological inhibition by Corylifol A. TAM; tumor-associated macrophage, TAOK1; Thousand-and-one kinase 1, TAOK3; Thousand-and-one kinase 3, Fn14; fibroblast growth factor inducible-14, TWEAK; Tumor necrosis factor-like weak inducer of apoptosis, NF-kB; nuclear factor kappa B, RELB; RELB proto-oncogene NF-kB subunit, FoxO3; forkhead box O3, MuRF1; muscle RING-finger protein 1, UPS; ubiquitin–proteasome system.

We propose an integrated model, illustrated schematically in Figure 4, in which PDA tumor cells and TAMs drive cachexia through two convergent arms: a directly demonstrated CCL2-TAM-CCL5-NF-kB-TWEAK-RELB-MuRF1/Atrogin-1 relay, and a muscle intrinsic TAOK1-p38-MAPK-FoxO3 arm activated by tumor-derived factors, in which the contribution from TWEAK/Fn14 signaling is biologically plausible but remains correlative. Both TAOK3 in the tumor and TAOK1 in muscle are pharmacologically druggable within this network, as demonstrated by the dual efficacy of Corylifol A [50], the precise linking of the two arms should be treated as a testable hypothesis for future integrated in vivo validation rather than an established mechanism.

## 7. Pharmacological Inhibitors

The multi-systemic, absolute reliance of PDA on TAOK3 for stemness, invasion, immune evasion, and the induction of cachexia positions these enzymes as compelling targets for precision oncology [70]. Recent high-throughput screening initiatives and structure-based drug design efforts have successfully yielded several potent chemical entities that demonstrate preclinical TAOK3 inhibitory efficacy summarized in Table 2 [40].

An important caveat to this pharmacological rationale is the likelihood of feedback reactivation and compensatory signaling upon TAOK3 inhibition. The intracellular cascades discussed above are richly interconnected; TAOK3 acts upstream of both the p38/MAPK and Hippo/YAP-TAZ axes and exerts inhibitory control over JNK/SAPK signaling, such that its blockade could plausibly disinhibit parallel MAPK branches. Furthermore, the inhibition of TAOK3 may also trigger compensatory receptor tyrosine kinase signaling, analogous to the well-documented feedback reactivation that limits the durability of MEK, ERK, and RAS-pathway inhibitors more broadly in KRAS-mutant PDA [17,71]. Similarly, the multi-step cachexia axis described in Section The CCL2-TWEAK-TAOK1 Feedforward Muscle Atrophy Loop involves several sequential paracrine and endocrine relays, any of which could in principle be bypassed by compensatory cytokine signaling if only a single node is inhibited. These considerations argue for combination strategies, and for explicit pharmacodynamic monitoring of parallel pathway activity as agents progress toward clinical evaluation.

### 7.1. Multi-Kinase Inhibition Strategies

Early pharmacological strategies aiming to intercept TAOK3 signaling primarily utilized broad-spectrum, multi-kinase inhibitors. The most prominent agent in this class is NCGC00188382, often referred to as Inhibitor #1. Originally synthesized with the intention of targeting the interleukin-2-inducible T-cell kinase (ITK), molecular profiling unexpectedly revealed that NCGC00188382 operates as a highly potent multi-kinase inhibitor, strongly suppressing the catalytic activity of TAOK3 in addition to inhibiting CDK7 and Aurora B kinases [56]. In advanced preclinical murine models of PDA, administration of NCGC00188382 yielded profound therapeutic effects. By inhibiting TAOK3, the compound disrupted the specific DDR dependency pathway relied upon by the aggressive CSC spheroids [53]. This precise disruption effectively bypassed the tumor defense G2/M cell cycle control mechanisms, leading directly to the near-complete eradication of the cancer stem cell population and yielding a suppression of distal metastasis in vivo [53].

### 7.2. Advanced Specific TAOK3 Inhibitors

The rapid evolution of structure-based virtual screening has catalyzed the discovery of highly refined small molecules engineered with extreme specificity for the unique ATP-binding pocket of TAOK3, minimizing the off-target toxicities associated with broader kinase inhibitors.

Discovered through the computational screening of a library containing over 10,000 lead-like compounds, Compound Z1 has emerged as a promising, specific TAOK3 inhibitor [40]. Extensive molecular dynamics simulations and molecular docking analyses confirmed that Compound Z1 successfully infiltrates the ATP-binding site, forming a highly rigid, exceptionally stable complex that essentially locks TAOK3 into a catalytically inert, inactive state [40]. Selectivity, however, remains a substantial challenge. When Compound Z1 was docked against the paralogous structure TAOK2 in complex with staurosporine (PDB: 2GCD), it displayed a predicted binding affinity within 0.3 kcal/mol of its affinity for TAOK3, indicating that the ATP-binding pocked is highly conserved across the TAOK family members and pocket-directed inhibitors are likely to have pan-TAOK inhibition [40].

Another highly potent and specific TAOK3 inhibitor, SBI-581, has repeatedly demonstrated therapeutic efficacy across diverse models of invasive carcinomas. In the context of the dense pancreatic stroma, the application of SBI-581 incapacitates the tumors invasive machinery [37]. This inhibitor depletes the population of highly motile, TKS5a-positive recycling endosomes, effectively halting the assembly of invadopodia [37]. Additionally, in esophageal SCC models, SBI-581 exhibits remarkable synergy with traditional cytotoxics. By actively disrupting the specific TAOK3-KMT2C-ETV5 signaling axis, SBI-581 suppresses the downstream transcription of the *IRGM* gene [41]. This blockade successfully paralyzes the tumors autophagic survival networks, effectively abolishing acquired cellular resistance and re-sensitizing the highly refractory cancer cells to cisplatin therapy [41].

### 7.3. Dual-Targeting Natural Bioactive

Perhaps the most conceptually innovative approach to dismantling the extensive TAOK network in PDA involves the deployment of specialized natural bioactives, most notably Corylifol A (CYA). CYA is a principal bioactive isoflavonoid extracted from the traditional medicinal plant *Psoralea corylifolia* [50]. Detailed molecular docking studies have demonstrated that CYA acts as a highly unique dual-inhibitor, directly engaging and binding to the kinase domains of both TAOK3 and TAOK1 [50].

The dual-action pharmacology of CYA provides a comprehensive therapeutic mechanism capable of simultaneously attacking the primary pancreatic tumor while preventing systemic cancer cachexia [50]. Within the hostile environment of the primary tumor bed, CYA readily permeates MIA PaCa-2 and PANC-1 pancreatic cancer cells and binds directly to TAOK3 [50]. The pharmacological blockade of TAOK3 by CYA lifts the established suppression of the JNK pathway by TAOK3. This inhibition triggers a massive, rapid elevation in the levels of phosphorylated JNK and cleaved Caspase-3, enforcing a profound cell cycle arrest strictly at the G1-phase and directing the malignant cells into terminal apoptosis [50].

CYA was also reportedly shown to directly bind to TAOK1, blocking its autophosphorylation [68]. This targeted inhibition interrupted the downstream activation of the skeletal muscle destructive p38-MAPK/FoxO3 axis [68].

The pharmacological targeting of the broader kinome in PDA, such as utilizing XL184 (cabozantinib) to inhibit the c-Met CSC marker [54], or deploying dual PI3K/mTOR inhibitors like GSK2126458 [70], has established a strong precedent for kinase-directed therapies. However, the unique, overarching integration of the TAO kinases into both the tumors internal survival mechanics and its external systemic destruction sets them apart. Combining highly specific TAOK3/TAOK1 inhibitors with standard cytotoxic regimens presents an opportunity to simultaneously debulk the primary tumor mass, eradicate the therapy-resistant CSC population, halt metastatic dissemination through the paralysis of invadopodia, and drastically improve overall patient survival and quality of life by mitigating the devastating effects of cachexia. However, as TAOK3 exhibits critical systemic physiological roles, including the maintenance of naïve T-cell survival via SHP-1 degradation, the future development of highly selective, tumor-targeted delivery systems will be paramount to maximize therapeutic efficacy and minimize off-target toxicity [60].

Several limitations of the current evidence base warrant acknowledgment. First, no TAOK3-selective inhibitor has entered clinical trials; all pharmacological agents addressed in Section 7 remain at the preclinical stage, and their pharmacokinetic, toxicological and clinical safety profiles in humans are unknown. Second, the cellular data underpinning the TAOK3 role in CSC maintenance and invasion derive predominantly from MIA PaCa-2 and PANC-1 cell lines, which do not capture the full transcriptomic and stromal heterogeneity of PDA. Third, the influence of TAOK3 on T-cell biology and the implication of its inhibition on normal adaptive immunity may potentially exacerbate the immunosuppression that PDA therapies seek to reverse.

## 8. Conclusions and Future Directions

As highlighted by comprehensive pan-cancer profiling, TAOK3 has emerged as a central signaling node potentially governing the lethality of PDA. By systematically orchestrating multiple recognized hallmarks of cancer, fortifying tumor-initiating stem cells via DDR, sustaining canonical TCR-signaling to preserve T-cell responsiveness, mediating M2-macrophage polarization to drive immune evasion and sustain the TME, directly modulating the Hippo/YAP/TAZ axis and establishing systemic cachexia, TAOK3 demands clinical attention.

The advent of chemical inhibitors with advanced 3D organoid screening platforms provides a compelling and robust preclinical foundation. Future translational research must focus on optimizing the pharmacokinetic profiles of these inhibitors and evaluating their synergy with both chemo- and immunotherapies. Ultimately, TAOK3 stands as a potential premier disease-modifying therapeutic target in pancreatic oncology.

## Figures and Tables

**Table 1:** Structural and physiological comparisons between TAOK subfamilies.

Structural Feature	TAOK1	TAOK2	TAOK3
Alternative nomenclature	MARKK, PSK2	PSK1	JIK, DPK
Chromosomal Location	17p11.2	16p11.2	12q24.23
Kinase Domain (N-Terminal)	Present (Reference Sequence)	Present (89.8% identity to TAOK1)	Present (88.6% identity to TAOK1; 82.7% to TAOK2)
Serine-Rich Domain	Present	Present	Present
Coiled-Coil Regions	Present	Present	Present
Leucine-Rich Repeat	Absent	Present	Absent

MARKK; Microtubule affinity-regulating kinase kinase, PSK; Prostate-derived Ste20-like kinase 2, JIK; JNK inhibitory kinase; TAOK1; Thousand-and-one kinase 1; TAOK2; Thousand-and-one kinase 2; TAOK3; Thousand-and-one kinase 3.

**Table 2:** Summary of dual-targeting bioactives and specific TAOK3 inhibitors reported in the literature with suggested mechanism of action and therapeutic efficacy.

Inhibitor Compound	Pharmacological Classification	Targeted Mechanism of Action	Therapeutic Efficacy	References
NCGC00188382	Multikinase Inhibitor (TAOK3, CDK7, Aurora B)	Disrupts tumor DDR synthetic dependency; overrides G2/M cell cycle checkpoint control	Near-complete eradication of CSC spheroids; vast suppression of PDA metastasis	[53]
Compound Z1	Specific TAOK3 Inhibitor	Forms rigid, stable complex via high-affinity ATP-pocket binding	Locks TAOK3 in inactive state	[40]
SBI-581	Specific TAOK3 Inhibitor	Arrests TKS5a vesicular trafficking; actively disrupts the oncogenic KMT2C-IRGM axis	Paralyses invadopodia assembly	[37]
Corylifol A (CYA)	Dual-Targeting Natural Isoflavonoid	Binds TAOK3 in tumor cells (activating JNK) and binds TAOK1 in skeletal muscle	Induces rapid PDA apoptosis; actively preserves host muscle mass by blocking FoxO3-UPS degradation	[50]
NCGC00188382	Multikinase Inhibitor (TAOK3, CDK7, Aurora B)	Overrides G2/M cell cycle checkpoint control	Near-complete eradication of CSC spheroids	[53]

DDR; DNA damage response, CDK; Cyclin-dependent kinase, CSC; cancer stem cell, TAOK3; Thousand-and-one kinase 3. ATP; adenosine triphosphate, TKS; Tyrosine kinase substrate, KMT2C; Lysine methyltransferase 2C, IRGM; Immunity-Related GTPase M, CYA; Corylifol A, JNK; c-Jun N-terminal kinase, TAOK1; Thousand-and-one kinase 1, PDA; pancreatic ductal adenocarcinoma.

## Data Availability

No new data were created or analyzed in this study. Data sharing is not applicable to this article.

## Abbreviations

The following abbreviations are used in this manuscript:

TAO	Thousand-and-one
TAOK3	Thousand-and-one kinase 3
PDA	Pancreatic ductal adenocarcinoma
KRAS	Kirsten rat sarcoma viral oncogene homolog
TME	Tumor microenvironment
MAPK	Mitogen-activated protein kinase
MAP3K	Mitogen-activated protein kinase kinase kinase
CSC	Cancer stem cell
TCR	T-cell receptor
PanIN	Pancreatic intraepithelial neoplasia
IPMN	Intraductal papillary mucinous neoplasm
5-FU	5-fluorouracil
DNA	Deoxyribose nucleic acid
GPCR	G-protein-coupled receptor
MST	Macrophage stimulating
LATS	Large tumor suppressor
YAP	Yes-associated protein
TAZ	Transcriptional co-activator with PDZ-binding motif
SAPK	Stress-Activated Protein Kinase
JNK	c-Jun NH_2_-terminal kinase
MARKK	Microtubule Affinity-Regulating Kinase Kinase
PSK	Prostate-Derived Ste20-Like Kinase 2
JIK	JNK Inhibitory Kinase
DPK	Double-stranded RNA-activated protein kinase-like
MEK	MAPK/ERK kinase
ATM	Ataxia telangiectasia mutated
CHEK	Checkpoint kinase
CDK	Cyclin-dependent kinase
PLK	Polo-like kinase 1
TGF	Transforming growth factor
SOX	SRY-box
LCK	Lymphocyte-specific protein tyrosine kinase
DDR	DNA damage response
MKK	Mitogen-activated protein kinase kinase
SHP	Src homology region 2 domain-containing phosphatase-1
TAM	Tumor-associated macrophage
TNF	Tumor necrosis factor
TWEAK	TNF-like weak inducer of apoptosis
Fn14	Fibroblast growth factor-inducible 14
MuRF	Muscle RING-Finger protein
FoxO3	Forkhead box O3
UPS	Ubiquitin–proteasome system
ITK	Interleukin-2-inducible T-cell kinase
KMT2C	Lysine methyltransferase 2C
ETV	ETS translocation variant
CYA	Corylifol A
MIA PaCa-2	MIAMI Pancreatic Carcinoma-2
